# Assessment of myocardial work in sarcomere gene mutation carriers, healthy controls and overt nonobstructive hypertrophic cardiomyopathy

**DOI:** 10.1186/s44156-025-00073-4

**Published:** 2025-02-26

**Authors:** Carla Marques Pires, George Joy, Miltiadis Triantafyllou, Ricardo Prista Monteiro, Ana Ferreira, Konstantinos Savvatis, Luis Rocha Lopes

**Affiliations:** 1 Department of Cardiology, Braga Hospital, Braga, Portugal; 2 Barts Heart Center, St Bartholomew’s Hospital, Barts Health NHS Trust, London, England; 3 Institute of Cardiovascular Science, University College London, London, England; 4 Halmstad Hospital, Region Halland, Halmstad, Sweden

**Keywords:** Nonobstructive hypertrophic cardiomyopathy, Carriers, Myocardial work, Sarcomere mutations

## Abstract

**Background:**

Hypertrophic cardiomyopathy (HCM) is defined by unexplained hypertrophy and often characterized by diastolic and systolic dysfunction. HCM patients are known to have impaired left ventricular (LV) myocardial work (MW), a more load-independent parameter compared to global longitudinal strain (GLS). We hypothesized that impaired MW might occur in sarcomere mutation carriers without LV hypertrophy.

**Methods and results:**

A single centre study with a case-control design. Patients with overt nonobstructive HCM and a causal sarcomere gene variant (*n* = 44), carriers (*n* = 51) and age and sex matched (to the carriers) healthy controls (*n* = 32) underwent a transthoracic echocardiogram including myocardial deformation analysis to calculate GLS and MW. Global work index (GWI) (1695 ± 332mmHg% vs. 1881.50 ± 490mmHg%, *p* = 0.001) and global constructive work (GCW) (2017.78 ± 323.05mmHg% vs. 2329.31 ± 485.44 mmHg%, *p* = 0.002) were lower in sarcomere mutation carriers compared to controls. LV ejection fraction and GLS were similar between these two groups. GWI (1209 ± 735mmHg% vs. 1695 ± 332mmhg%, *p* < 0.001), GCW (1456 ± 703mmHg% vs. 1993 ± 389mmHg%, *p* < 0.001), global wasted work (GWW) (117 ± 148mmHg% vs. 96 ± 69mmHg%, *p* = 0.006) and global work efficiency (GWE) (89 ± 7% vs. 95 ± 3%, *p* < 0.001)] were worse in overt non-obstructive HCM patients.

**Conclusion:**

We show for the first time that MW indexes were significantly worse in sarcomere mutation carriers compared to controls, suggesting that MW is more sensitive to early changes than GLS and could have a significant role in the evaluation and follow-up of carriers.

## Introduction

Hypertrophic cardiomyopathy (HCM) is a myocardial disease defined by left ventricle hypertrophy (LVH) in any myocardial segment, that is not solely explained by abnormal loading conditions [1]. 

In up to 40% of cases, a pathogenic or likely pathogenic sarcomeric gene variant is identified. In most cases, this disease is inherited as an autosomal dominant trait. The two most common causal genes are β-Myosin Heavy chain (*MYH7*) and Myosin-binding Protein C (*MYBPC3*), identified in 70% of genotype-positive patients [1, 2]. 

HCM is characterized by distinctive myocardial histological changes: myocyte hypertrophy, myofibrillar disarray, microvascular dysplasia and myocardial fibrosis. These ultimately lead to small and stiff ventricles with impaired diastolic and systolic function despite preserved ejection fraction (EF) [2, 3]. Previous studies described a reduced left ventricle (LV) global longitudinal strain (GLS) despite normal or supranormal EF, reflecting the presence of systolic dysfunction due to impaired longitudinal function [4]. Additionally, abnormal LV-GLS has been correlated with myocardial fibrosis and adverse composite cardiac outcomes [5]. 

Nevertheless, GLS is an imperfect tool in this population due to pre and after-load dependency. Myocardial work (MW) is a more load-independent tool and a novel noninvasive approach to evaluate myocardial performance. MW integrates LV deformation and afterload by constructing a pressure-strain loop. Therefore, it enables investigation of LV performance in cases of changes in afterload, which could lead to misleading conclusions if relying solely on strain analysis [6, 7]. Recent studies have shown that reduced MW in patients with overt HCM correlated with myocardial fibrosis and worst prognosis [8, 9]. 

Regarding sarcomere mutation carriers, male gender and electrocardiographic anomalies were associated with higher risk of developing overt HCM in recent work [10]. In contrast, neither EF nor LV-GLS had clear prognostic value for the development of HCM during follow-up [11]. 

MW was never studied in sarcomere mutation carriers. The aim of this study is to assess MW in this population and to compare it with healthy controls and overt HCM patients.

## Methods

### Study population

This was a single-center study with a case-control design developed at the Inherited Cardiovascular Disease Unit, Barts Heart Center, St Bartholomew’s Hospital, Barts Health NHS Trust. Ethical approval for the study was given by the Research Ethics Committee (IRAS 227168) and met the criteria established by the Declaration of Helsinki [12]. All participants provided written informed consent.

The study population comprised three groups: group 1 was composed of carriers of a likely pathogenic or pathogenic sarcomere gene variant; group 2 included patients with overt nonobstructive HCM (NOHCM) with a likely pathogenic or pathogenic sarcomere gene variant and group 3 were age and sex-matched (to the carriers) healthy controls. NOHCM was defined as peak Doppler LV outflow tract gradient of < 30 mmHg, at rest and Valsalva [1]. 

Adult individuals with subclinical or overt HCM were prospectively recruited from databases for genotyped patients. Pathogenicity for detected variants was assessed using American College of Medical Genetics (ACMG) criteria [13]. 

Inclusion criteria for the current study were as follows (i) overt NOHCM were diagnosed as per the guidelines (myocardial wall thickness equal or greater than 15 mm in any cardiac segment by any imaging modality or equal or greater than 13 mm in individuals with a first degree relative with confirmed HCM); [1, 2] (ii) subclinical HCM (Genotype + LVH-) were individuals with pathogenic/likely pathogenic variants confirmed on cascade screening but with myocardial wall thickness less than 13 mm; (iii) healthy volunteers with no relevant past medical history or risk factors for coronary disease were prospectively matched for age and sex to the subclinical HCM (Genotype + LVH-) cohort.

## Echocardiography

All participants underwent a transthoracic echocardiogram including myocardial deformation analysis to calculate GLS and MW.

LV measurements and left atrium (LA) measurements were routinely obtained. Measurements of mitral inflow (E wave-early diastolic wave and A wave-late diastolic wave) and e′ velocity were obtained from the apical 4-chamber view using pulsed-wave Doppler (PWD) and tissue Doppler imaging (TDI), respectively. LVEF was calculated using biplane Simpson’s rule [14]. LV GLS and MW were measured using the speckle tracking technique.

The same investigator, blinded to case-control status, reviewed the images, and performed offline myocardial deformation analysis in all study subjects.

The offline analysis was done on *EchoPac* V203 (*General Electric*^®^*Healthcare*). Two-dimensional LV images from apical four, two and three-chambers were acquired with frame rates between 40 and 80 frames/s. The software automatically outlined the LV endocardium and the tracking was carefully observed and manually adjusted when necessary. Similarly, to previous studies [6], non-invasive brachial artery blood pressure evaluation was used to replace LV systolic pressure. This measurement was performed in the same-day, prior to the realization of the transthoracic echocardiogram. A pressure-strain loop (PSL) and resultant MW parameters were automatically obtained.

Global work index (GWI) reflected the MW performed by the LV during systole and was the area of PSL from mitral valve closure to mitral valve opening. Global constructive work (GCW) was the MW used for shortening (positive work) during systole and lengthening (negative work) during isovolumetric relaxation. Global wasted work (GWW) was the MW used for lengthening (negative work) during systole and shortening (positive work) during isovolumetric relaxation. Finally, global work efficiency (GWE) was the ratio between GCW and total MW (sum of GCW and GWW).

### Statistical analysis

Continuous variables were tested for normality using Kolmogorov-Smirnov’s test. Measures of central tendency (mean or median) and dispersion (standard deviation or interquartile range) were chosen according to normality test result. To examine differences between groups in normality distributed variables the t-student test or Welsh test (if homogeneity of variances was not assumed) was used. In non-normality distributed variables, Mann–Whitney test was performed. Categorical variables were expressed as relative and absolute frequencies and compared using the Chi-square test (χ2) or Fisher’s test.

All analyses used *IBM SPSS*^®^ (version 26; *IBM corp.*,* Armonk*,* NY*). Statistical significance was defined when *p* < 0.05.

## RESULTS

This study included 95 patients with a likely pathogenic or pathogenic sarcomere gene variant: 51 carriers (group 1) and 44 with overt NOHCM (group 2). Baseline characteristics of study subjects are presented in Table 1.

**Table 1:** Baseline clinical data of group 1 and group 2 participants

	G1: Carriers *n* = 51	G2: Overt NOHCM *n* = 44	*p*-value
**Baseline features**			
Age, years, mean (SD)	39.29 (11.86)	52.04 (13.32)	< 0.001
Female, n (%)	30.00 (58.82)	17.00 (33.33)	0.01
BMI, kg/m2, median (IQ)	23.56 (6.48)	25.78 (7.15)	0.004
BSA, mean (SD)	1.85 (0.20)	1.98 (0.24)	0.005
Sarcomere gene variant, n (%)			0.581
*MYBPC3*	31.00 (60.78)	36.00 (70.59)	
*MYH7*	12.00 (23.53)	9.00 (17.65)	
Other	8.00 (15.69)	6.00 (11.76)	
Family history of sudden death, n(%)	27.00 (52.94)	19.00 (37.25)	0.108
Arterial hypertension, n(%)	5.00 (9.80)	21.00 (41.18)	< 0.001
Diabetes, n (%)	1.00 (1.96)	8.00 (15.69)	0.015
Dyslipidemia, n (%)	4.00 (7.84)	15.00 (29.41)	0.005
Smoking, n(%)	5.00 (9.80)	8.00 (15.69)	0.353
Other cardiac disease, n(%)	4.00 (7.84)	8.00 (15.69)	0.218

BMI: Body mass index; BSA: Body surface area; IQ: Interquartile Range; *MYBPC3*: Cardiac myosin-binding protein C3; *MYH7*: myosin heavy chain 7; NOHCM: Nonobstructive hypertrophic cardiomyopathy, SD: standard deviation

Compared with carriers, overt NOHCM patients were older, had a lower prevalence of female gender, higher values of body mass index and a higher prevalence of cardiovascular risk factors. We found no significant differences regarding family history of sudden death. *MYBPC3* was the most frequent causal gene in both groups.

Regarding transthoracic echocardiogram evaluation (Table 2) patients with overt NOHCM had higher values of E/e’ ratio and lower values of MAPSE. LV dimensions, TAPSE and LVEF did not differ significantly between groups.

**Table 2:** Echocardiographic features of group 1 and group 2 participants

	G1: Carriers *n* = 51	G2: Overt NOHCM *n* = 44	*p*-value
**Transthoracic echocardiogram: routine evaluation**			
EDLVD, mm, median (IQ)	44.00 (8.00)	44.30 (7.60)	0.274
ESLVD, mm, median (IQ)	28.85 (7.00)	29.75 (7.70)	0.962
MWT, mm, median (IQ)	10.00 (2.00)	17.00 (3.00)	< 0.001
LA diameter, mm, median (IQ)	33.00 (6.00)	42.50 (6.00)	< 0.001
LA area, cm², median (IQ)	18.00 (5.30)	23.60 (8.10)	< 0.001
LA volume/BSA, ml/m², median (IQ)	24.32 (12.94)	37.47 (22.64)	< 0.001
LVEF, %, mean (SD)	59.76 (5.11)	61.07 (8.62)	0.381
MAPSE, mm, median (IQ)	14.50 (3.00)	9.10 (4.30)	< 0.001
TAPSE, mm, mean (SD)	23.34 (4.05)	22.08 (5.43)	0.198
E/e’, median (IQ)	5.85 (2.48)	8.62 (4.66)	< 0.001
**Blood pressure**			
Systolic arterial BP, mmHg, median (IQ)	115.00 (20.00)	120.50 (24.00)	0.017
Diastolic arterial BP, mmHg, median (IQ)	70.00 (12.00)	73.50 (12.00)	0.110
**Myocardial deformation analysis**			
GWI, mmHg%, median (IQ)	1695.00 (332.00)	1229 0.50 (474.64)	< 0.001
GCW, mmHg%, mean (SD)	2017.78 (323.05)	1545.08 (537.71)	< 0.001
GWW, mmHg%, median (IQ)	96.00 (69.00)	116.00 (158.00)	0.028
GWE, %, median (IQ)	95.00 (3.00)	89.00 (8.00)	< 0.001
GLS, %, median (IQ)	-18.00 (3.00)	-13.00 (4.00)	< 0.001

BSA: Body surface area; BP: Blood pressure; EDLVD: end-diastolic left ventricular diameter; ESLVD: end-systolic left ventricular diameter; IQ: interquartile range; GWI: Global work index; GCW: Global constructive work; GWW: Global wasted work; GWE: Global work efficiency; GLS: Global longitudinal strain; LA: Left atrium; LVEF: left ventricle ejection fraction; MAPSE: mitral annular plane systolic excursion; MWT: Maximum wall thickness; NOHCM: Nonobstructive hypertrophic cardiomyopathy, SD: standard deviation, TAPSE: tricuspid annular plane systolic excursion

**Fig. 1:**
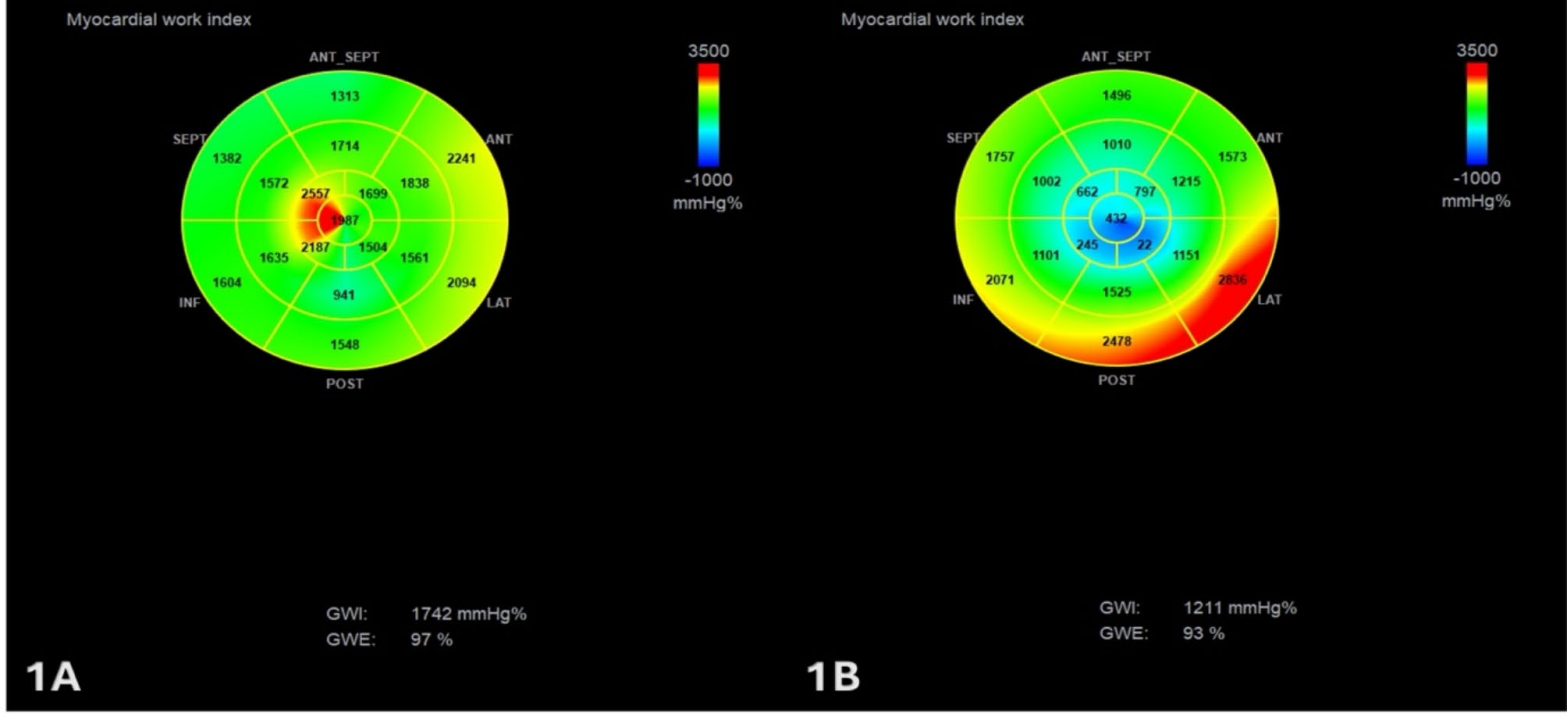
Representative example of myocardial work parameter plot and pressure-strain loop in one mutation carrier (1 **A**) and one patient with overt nonobstructive hypertrophic cardiomyopathy (1**B**)

**Fig. 2:**
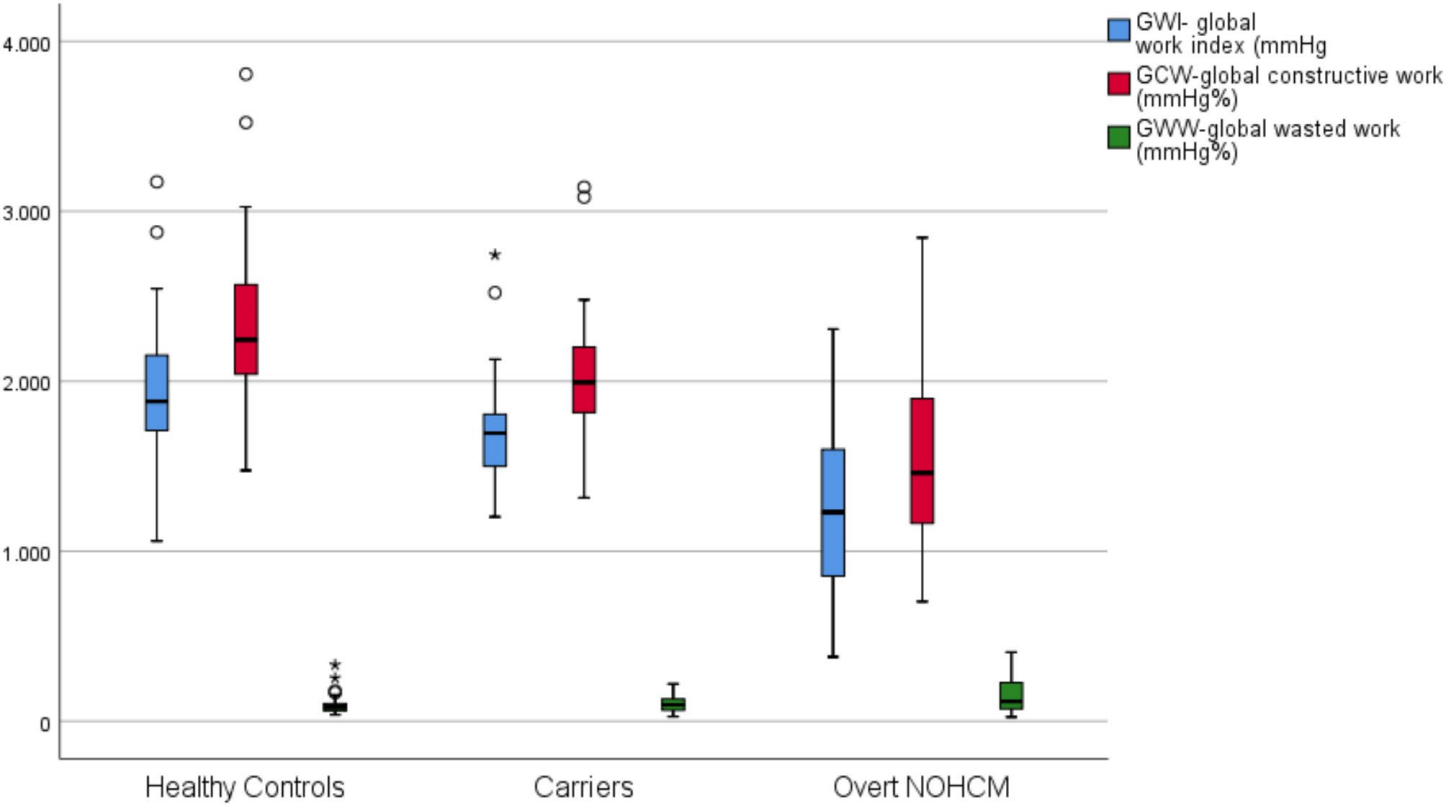
Box plot comparing GWI, GCW and GWW among participants of this study GWI: Global work index; GCW: Global constructive work; GWW: Global wasted work

**Fig. 3:**
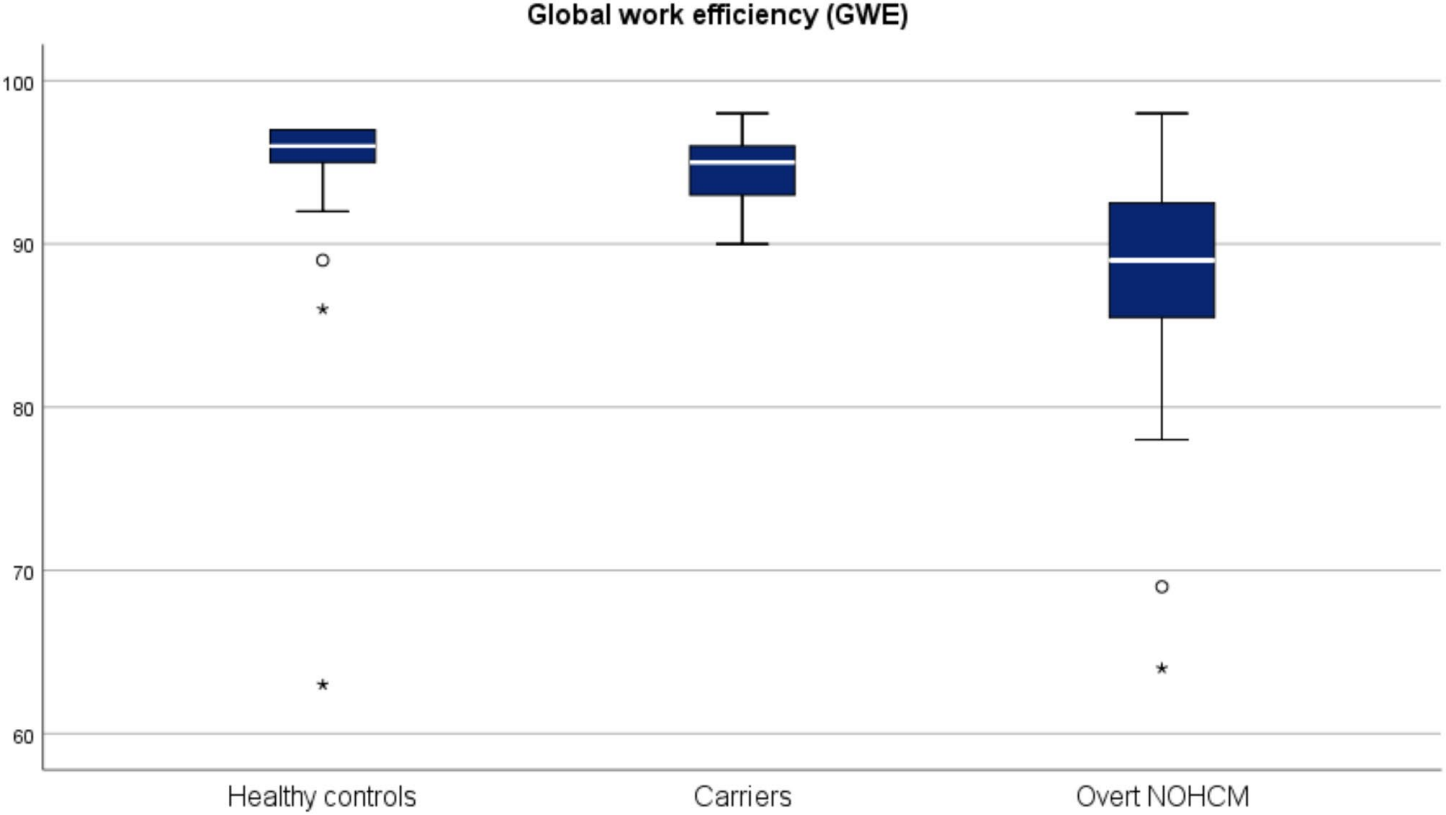
Box plot comparing GWE among participants of this study GWE: Global work efficiency

Myocardial deformation analysis revealed that LVGLS and all MW indexes (GWI: 1230vs1695mmHg%, *p* < 0.001; GCW 1545vs2018mmHg%, *p* < 0.001, GWW 116vs96mmHg%, *p* = 0.028; GWE 89vs95%, *p* < 0.001) were significantly worse in overt NOHCM patients (Figs. 1A, B, 2 and 3).

Further description of echocardiographic features is presented in Table 2.

Baseline demographic characteristics of the controls are presented in Table 3.

**Table 3:** Baseline clinical data of group 1 and group 3 participants

	G1: Carriers *n* = 51	G3: controls *n* = 32	*p*-value
**Baseline features**			
Age, years, mean (SD)	39.29 (11.86)	41.69 (15.00)	0.447
Female, n (%)	30.00 (58.82)	18.00 (56.25)	0.817
BSA, mean (SD)	1.85 (0.20)	1.87 (0.25)	0.708

BSA: Body surface area; SD: standard deviation

There were no differences regarding LVEF, E/e’ ratio and LVGLS between carriers and controls. However, carriers had significantly lower values of GWI (1695vs1882mmHg%, *p* = 0.001) and GCW (2018vs2329mmHg%, *p* = 0.002) - Fig. 2.

Further description of echocardiographic features is presented in Table 4.

**Table 4:** Echocardiographic features of group 1 and group 3 participants

	G1: Carriers *n* = 51	G3: Controls *n* = 32	*p*-value
**Transthoracic echocardiogram**			
EDLVD, mm, median (IQ)	44.00 (8.00)	51.50 (7.80)	< 0.001
ESLVD, mm, mean (SD)	28.66 (4.36)	33.25 (4.47)	< 0.001
LA area, cm², mean (SD)	17.72 (4.33)	18.56 (3.51)	0.360
2D LVEF, %, mean (SD)	59.76 (5.11)	58.81 (3.69)	0.960
E/e’, median (IQ)	5.86 (2.00)	5.68 (2.00)	0.244
**Blood pressure**			
Systolic arterial BP, mmHg, median (IQ)	115.00 (20.00)	123.00 (26.00)	0.09
Diastolic arterial BP, mmHg, median (IQ)	70.00 (12.00)	74.50 (14.00)	0.101
**Myocardial deformation analysis**			
GWI, mmHg%, median (IQ)	1695.00 (332.00)	1881.50 (490.00)	0.001
GCW, mmHg%, mean (SD)	2017.78 (323.05)	2329.31 (485.44)	0.002
GWW, mmHg%, median (IQ)	96.00 (69.00)	81.50 (43.00)	0.457
GWE, %, median (IQ)	95.00 (3.00)	96.00 (2.00)	0.177
GLS, %, median (IQ)	-18.00 (3.00)	-18.45 (2.60)	0.256

BP: Blood pressure; EDLVD: end-diastolic left ventricular diameter; ESLVD: end-systolic left ventricular diameter; IQ: interquartile range; GWI: Global work index; GCW: Global constructive work; GWW: Global wasted work; GWE: Global work efficiency; GLS: Global longitudinal strain; LA: Left atrium; LVEF: left ventricle ejection fraction, SD: standard deviation

Patients with *MYBPC3 vs MYH7* gene variants did not have significantly different MW indexes and LVGLS in subclinical (GWI: 1689vs1753mmHg%, *p* = 0.623; GCW 2027vs2057mmHg%, *p* = 0.844, GWW 103vs86mmHg%, *p* = 0.277; GWE 95vs96%, *p* = 0.314) or overt NOHCM (GWI: 1225vs1249mmHg%, *p* = 0.901; GCW 1556vs1498mmHg%, *p* = 0.783, GWW 116vs141mmHg%, *p* = 0.959; GWE 91vs88%, *p* = 0.401) (Tables 5 and 6).

**Table 5:** Myocardial deformation analysis stratified by sarcomere gene in group 1

	Carriers with *MYBPC3* *n* = 31	Carriers with *MYH7* *n* = 12	*p*-value
**Blood pressure**			
Systolic arterial BP, mmHg, mean (SD)	117.61 (15.22)	118.83 (19.24)	0.846
Diastolic arterial BP, mmHg, median (IQ)	70.00 (15.00)	70.00 (11.00)	0.530
**Myocardial deformation analysis**			
GWI, mmHg%, mean (SD)	1689.13 (239.38)	1753.17 (415.30)	0.623
GCW, mmHg%, mean (SD)	2027.03 (280.34)	2056.83 (483.14)	0.844
GWW, mmHg%, median (IQ)	103.13 (49.38)	85.58 (39.27)	0.277
GWE, %, median (IQ)	95.00 (3)	95.50 (3)	0.314
GLS, %, median (IQ)	-18.00 (2.00)	-18.50 (4.50)	0.565

BP: Blood pressure; IQ: interquartile range; GWI: Global work index; GCW: Global constructive work; GWW: Global wasted work; GWE: Global work efficiency; GLS: Global longitudinal strain, SD: standard deviation

**Table 6:** Myocardial deformation analysis stratified bysarcomere gene in group 2

		Overt NOHCM + MYBPC3 *n* = 31	Overt NOHCM + MYH7 *n* = 8	*p*-value
**Blood pressure**				
Systolic arterial BP, mmHg, mean (SD)		122.00 (20.00)	119.00 (33.00)	0.142
Diastolic arterial BP, mmHg, mean (SD)		75.45 (9.49)	71.75 (8.91)	0.326
**Myocardial deformation analysis**				
GWI, mmHg%, mean (SD)		1224.87 (456.91)	1248.75 (571.27)	0.901
GCW, mmHg%, mean (SD)		1555.97 (510.40)	1497.75 (598.89)	0.783
GWW, mmHg%, median (IQ)		116.00 (164.00)	141.00 (170.00)	0.959
GWE, %, median (IQ)		91.00 (8.00)	88.00 (13.00)	0.401
GLS, %, mean (SD)		-12.81 (3.24)	-13.37 (4.75)	0.691

BP: Blood pressure; IQ: interquartile range; GWI: Global work index; GCW: Global constructive work; GWW: Global wasted work; GWE: Global work efficiency; GLS: Global longitudinal strain, NOHCM: Nonobstructive hypertrophic cardiomyopathy, SD: standard deviation

## Discussion

The main goal of this study was to evaluate MW in sarcomere carriers for the first time and assess whether an abnormal MW is part of the constellation of findings characteristic of subclinical HCM in mutation carriers.

HCM phenotype encompasses morphological and functional abnormalities beyond hypertrophy. Subclinical features occur early in carriers, including electrocardiographic abnormalities (e.g. repolarization changes), multiple myocardial crypts, hyperdynamic radial LV function but with lower GLS, long mitral leaflets and displacement of the papillary muscles [15–18]. 

In our study, carriers had significantly worse MW indexes: lower values of GWI (1695 ± 332mmHg% vs. 18881.50 ± 490mmHg%, *p* = 0.001) and GCW (2017.78 ± 323.05mmHg% vs. 2329.31 ± 485.44 mmHg%, *p* = 0.002). There were no differences of LVEF and LVGLS between carriers and controls [11].

Recent studies assessed MW in overt HCM (8–9, 15). Galli et al. [8]. in 2019, evaluated 82 patients with non-obstructive HCM and found a significantly reduced GCW, which was associated with myocardial fibrosis assessed by cardiac magnetic resonance [8]. In line with Russel et al. [6], these authors suggested that abnormal MW indexes reflected changes in myocardial oxygen consumption and metabolism, secondary to disarray and microvascular dysfunction. These findings were also supported by Brás et al. [19] who have shown that MW was significantly correlated with the extent of ischemia in cardiac magnetic resonance, independently of left ventricular hypertrophy or fibrosis.

Moreover, Gonçalves et al. [20] in 2021, described that GCW and GWI were significantly correlated with late gadolinium enhancement and a cut-off less than or equal to 1550 mmHg% of GCW was associated with significant fibrosis on cardiac magnetic resonance with a sensitivity of 91% and a specificity of 76%. Furthermore, Hiemstra et al. [9] demonstrated that in patients with non-obstructive HCM, a GCW greater than 1730 mmHg% was associated with better event-free survival.

Our study suggests that MW is more sensitive to early changes than LVGLS. These findings could have a significant implication for the evaluation and follow-up of sarcomere mutation carriers, since one of the possible future applications of identifying early markers of the disease would be an optimized tailoring of relatives under follow-up.

A possible explanation to our findings is that abnormal MW indexes in carriers reflect impaired myocardial perfusion and tissue changes (disarray) that occurs before LV hypertrophy development. Recent studies had already challenged the conventional view, in which perfusion defects would be only secondary to LV hypertrophy and subsequent extravascular compressive forces and elevated intraventricular pressures. Hughes et al. [21] evaluated 50 individuals with genotype -positive and LVH-negative using perfusion mapping cardiac magnetic resonance and reported that myocardial perfusion reserve was lower in carriers with a subendocardial: subepicardial myocardial perfusion reserve gradient. Vigneault et al. [22], in a feature-tracking cardiac magnetic resonance study, described that mutation carriers had higher circumferential transmural strain difference than healthy controls, reflecting underlying myocardial dysfunction before left ventricle hypertrophy. Joy et al. [23], using diffusion tensor imaging, have shown that microstructural changes and microvascular disease occur in the absence of LV hypertrophy in sarcomere mutation carriers.

In an era of emerging therapies with potential for disease modification, myocardial work could be tested as an early disease marker in future trials, to help select the individuals that would benefit most and also access the response to those therapies.

### Limitations

Our study is a single-center and single vendor study with case-control design and a relatively small sample. Larger studies with prospective design should be performed to assess the prognostic implications of our results, including in integration with other known markers of early disease, derived from ECG and cardiac magnetic resonance.

In addition, participants of group 1 (overt NOHCM) have an asymmetrical hypertrophy which can impact strain and MW analysis since this algorithm assumes that LV wall thickness is equal across all myocardial segments. However, these parameters have already been used in multiple studies in patients with HCM [8, 9, 19, 20]. Our main focus and novelty is the identification of early MW changes in carriers, where hypertrophy is not present.

## Conclusion

In summary, our study showed that MW is abnormal in HCM mutation carriers and part of the subclinical HCM phenotype.

## Data Availability

The datasets used and/or analysed during the current study are available from the corresponding author on reasonable request.

## Abbreviations

EF: Ejection fraction
GLS: Global longitudinal strain
GCW: Global constructive work
GWE: Global work efficiency
GWI: Global work index
GWW: Global wasted work
HCM: Hypertrophic cardiomyopathy
LA: Left atrium
LV: Left ventricle
LVH: Left ventricle hypertrophy
MAPSE: Mitral annular plane systolic excursion
MYH7: β-Myosin Heavy chain
MYBPC3: Myosin-binding Protein C
MW: Myocardial work
NOHCM: Nonobstructive Hypertrophic cardiomyopathy
PSL: Pressure-strain loop
PWD: Pulsed-wave Doppler
TAPSE: Tricuspid annular plane systolic excursion
TDI: Doppler imaging
